# Stratified Abstraction of Access Control Policies

**DOI:** 10.1007/978-3-030-53288-8_9

**Published:** 2020-06-13

**Authors:** John Backes, Ulises Berrueco, Tyler Bray, Daniel Brim, Byron Cook, Andrew Gacek, Ranjit Jhala, Kasper Luckow, Sean McLaughlin, Madhav Menon, Daniel Peebles, Ujjwal Pugalia, Neha Rungta, Cole Schlesinger, Adam Schodde, Anvesh Tanuku, Carsten Varming, Deepa Viswanathan

**Affiliations:** 8 Microsoft Research Lab, Redmond, WA USA; 9 University of Southern California, Los Angeles, CA USA; Amazon Web Services, Seattle, USA

## Abstract

The shift to cloud-based APIs has made application security critically depend on understanding and reasoning about *policies* that regulate access to cloud resources. We present *stratified predicate abstraction*, a new approach that summarizes complex security policies into a compact set of positive and declarative statements that precisely state *who* has access to a resource. We have implemented stratified abstraction and deployed it as the engine powering AWS’s IAM Access Analyzer service, and hence, demonstrate how formal methods and SMT can be used for security policy *explanation* .

## Introduction

A growing number of developers are using cloud-based implementations of basic resources like associative arrays, encryption, storage, queuing, and event-driven execution, to engineer client applications. For example, millions of Amazon Web Services (AWS) customers use cloud APIs like Amazon SQS for queues, Amazon S3 for storage, AWS KMS for crypto key management, Amazon DynamoDB for associative arrays, and AWS Lambda for executing functions in a pure virtualized environment. This shift to the cloud has made application security critically depend upon deeply understanding and reasoning about *policies* that regulate how different principals are allowed to access cloud resources. AWS users, for example, configure principals in the Identity and Access Management (IAM) service. The users define which requests are allowed access via *resource policies* which allow some resources to be purposefully shared with the entire internet, while restricting access to others to limited sets of identities.

The IAM policy language has many features that are essential to allow users to build a wide array of possible applications. Some of these features make reasoning about policies challenging. First, individual policy elements can use regular expressions, negation, and conditionals. Second, the policy elements can interact with each other in subtle ways that make the net effect of a policy unclear. Previously, we developed Zelkova [2], a tool that encodes policies as logical formulas and then uses SMT solvers [3, 8] to answer questions about policies, *e.g.* whether a particular policy is *correct*, too strict, or too permissive. While Zelkova can be queried to *explore* the properties of policies *e.g.* whether some resource is “publicly” accessible, our experience shows that formal policy analysis remains challenging as users must have sufficient technical sophistication to realize the criteria important to them *and* be able to formalize the above as Zelkova queries.

**Fig. 1.**
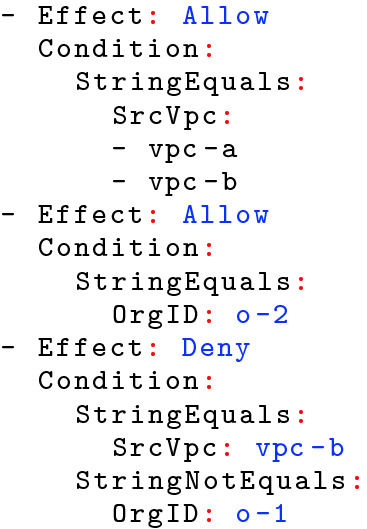
An example AWS policy

**Fig. 2.**
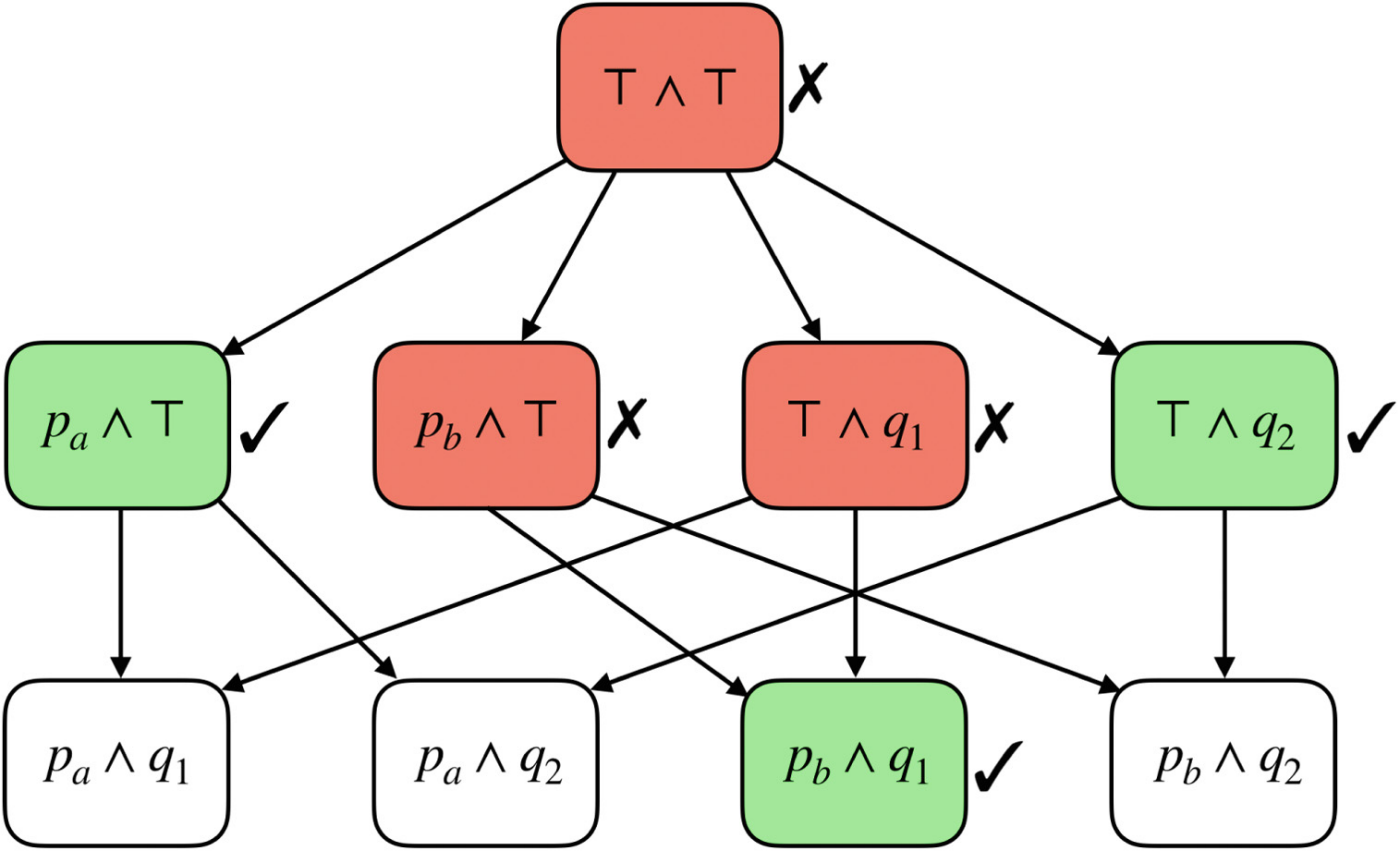
Stratified abstraction search tree

In this paper, we present a new approach to help users understand whether their policy is correct, by *abstracting* the policy into a compact set of positive and declarative statements that precisely summarize *who* has access to a resource. Users can review the summary to decide whether the policy grants access according to their intentions. The key challenge to computing such summaries is the combinatorial blowup in the number of possible requests, which comprise the combination of user name and account, identifiers, hostnames, IP addresses and so on. Our key insight is that we can make summarization tractable via *stratified predicate abstraction*, which allows us to collapse many equivalent (concrete) requests into a single (abstract) *finding*. To this end, we introduce a new algorithm for computing stratified abstractions of policies, yielding a set of findings that are *sound*, *i.e.* which include all possible requests that can be granted access, and *precise*, *i.e.* where the findings are as specific as possible.

We have implemented stratified abstraction and deployed it as the engine powering AWS’s recently launched IAM Access Analyzer service, which helps users reason about the semantics of their policy configurations. We present an empirical evaluation of our method over a large set of real-world IAM policies. We show that IAM Access Analyzer generates a sound, precise, and *compact* set of findings for complex policies, taking less than a second per finding. Thus, our results show how key ideas like SMT solving and predicate abstraction [1, 5], can be used not just to *verify* computing systems, but to precisely *explain* their behavior to users.

## Overview

AWS access control policies specify *who* has access to a given resource, via a set of Allow and Deny statements that grant and prohibit access, respectively. Figure 1 shows a simplified policy specifying access to a particular resource. This policy uses conditions based on which network (known as a VPC) the request originated from and which organizational Amazon customer (referred to by an Org ID) made the request. The first statement *allows* access to any request whose SrcVpc is either vpc-a *or* vpc-b. The second statement *allows* access to any request whose OrgId is o-2. However, the third statement *denies* access from vpc-b *unless* the OrgId is o-1.

Crucially, for each request, access is granted only if: (a) *some* Allow statement matches the request, and (b) *none* of the Deny statements match the request. Consequently, it can be quite tricky to determine what accesses are allowed by a given policy. First, individual statements can use regular expressions, negation, and conditionals. Second, to know the effect of an allow statement, one must consider all possible deny statements that can *overlap* with it, *i.e.* can refer to the same request as the allow. Thus, policy verification is not *compositional*, in that we cannot determine if a policy is “correct” simply by *locally* checking that each statement is “correct”. Instead, we require a *global* verification mechanism, that simultaneously considers all the statements and their subtle interactions, to determine if a policy grants only the intended access.

As policies organically grow and become more complex and baroque, the ultimate question that users have is: “is my policy correct?” Of course, this *specification* problem has bedeviled formal methods from the day they were invented. In our context: how does the security analyst know whether the policy is, in fact not too strict or too permissive? Zelkova [2] is already used by users of Amazon’s Simple Storage Service (S3) to determine whether any of their “data buckets” are *publicly* accessible. More generally, the AWS Config service provides templated Zelkova checks that can be filled in by users to validate their policies. Some advanced users even use the Zelkova service directly, asking their own questions about policies. While all of the above are useful, formal policies and formal analysis remains difficult to use, as the user must have sufficient technical sophistication to: (1) *intuit* the criteria important to them, (2) *formalize* the above in the query language of Zelkova, and (3) *interpret* the results returned by the tool. Ultimately, to answer “is this policy correct?”, the tool must *help the user understand* what “correct” means in their particular context.

### Approach

The core contribution of this work is to change the question from *“is this policy correct?”* to *“who has access?”*. The response to the former is a Boolean while the response to the latter is a set of *findings*. There are several key requirements that findings must meet to be useful in the context of analyzing security policies and answering the question *“who has access?”*.

**Sound.** Users need confidence that findings *summarize* a policy. In particular, we must ensure that *every* access allowed by the policy is represented by *some* finding. This over-approximation crucially enables compositional reasoning about the policy: if a user deems that *each* finding is safe, then she may rest assured that the *entire* policy is safe.

**Precise.** Users require that findings be *specific*. A finding of “everybody has access” is a sound and over-approximate summary of every policy, but is only useful if the policy allows everyone access. Instead, we want findings that adhere closely to the accesses allowed by the policy, and do not report false-alarms that say certain identities have access when that is not, in fact, the case.

**Compact.** Users require that the set of findings be *small*. For example, we could simply *enumerate* all the different kinds of requests that have access, but such a list would typically be far too large to manually inspect. Instead, we require that the findings be a compact representation of who has access, while still ensuring soundness and precision.

***Example.*** For example, the policy in Fig. 1 can be summarized through a set of three findings, that say that access is granted to a request iff:

- Its SrcVpc is vpc-a, *or*,
- Its OrgId is o-2, or,
- Its SrcVpc is vpc-b *and* its OrgId is o-1.

The findings are sound as no other requests are granted access. The findings are precise as in each case, there are requests matching the conditions that are granted access.^1^ Finally, the findings compactly summarize the policy in three positive statements declaring *who* has access.

### Solution: Computing Findings via Stratified Abstraction

Next, we describe an informal overview of our algorithm for computing the findings, by building it up in three stages.

***1: Concrete Enumeration.*** One approach to synthesize findings would be to (1) *enumerate* possible requests, (2) *query* Zelkova to filter out the requests that do not have access, and (3) *return* the remainder as findings. Such an approach is guaranteed to be both sound and precise. However, real-world policies comprise many fields, each of which have many possible values. For example, there are $10^{12}$ (currently) possible AWS account numbers and $2^{128}$ possible IPv6 addresses. Enumerating all possible requests is computationally *intractable*, and even if it were, the resulting set of findings is far too large and hence *useless*.

***2: Predicate Abstraction.*** We tackle the problem of summarizing the super-astronomical request-space by using *predicate abstraction*. Specifically, we make a syntactic pass over the policy to extract the set of constants that are used to constrain access, and we use those constants to generate a family of predicates whose conjunctions compactly describe partitions of the space of all requests. For example, from the policy in Fig. 1 we would extract the following predicates

$$ \begin{array}{lll} p_a \doteq \mathsf {SrcVpc} = \mathtt {\mathtt {vpc}\text{- }\mathtt {a}},\ &{} p_b \doteq \mathsf {SrcVpc} = \mathtt {\mathtt {vpc}\text{- }\mathtt {b}},\ &{} p_\star \doteq \mathsf {SrcVpc} = \mathtt {\star },\\ q_1 \doteq \mathsf {OrgId} = \mathtt {\mathtt {o}\text{- }\mathtt {1}},\ &{} q_2 \doteq \mathsf {OrgId} = \mathtt {\mathtt {o}\text{- }\mathtt {2}},\ &{} q_\star \doteq \mathsf {OrgId} = \mathtt {\star }. \end{array} $$

The first row has three predicates describing the possible value of the SrcVpc of the request: that it equals $\mathtt {vpc}\text{- }\mathtt {a}$ or $\mathtt {vpc}\text{- }\mathtt {b}$ or some value other than $\mathtt {vpc}\text{- }\mathtt {a}$ and $\mathtt {vpc}\text{- }\mathtt {b}$. Similarly, the second row has three predicates describing the value of the OrgId of the request: that it equals $\mathtt {o}\text{- }\mathtt {1}$ or $\mathtt {o}\text{- }\mathtt {2}$ or some value other than $\mathtt {o}\text{- }\mathtt {1}$ and $\mathtt {o}\text{- }\mathtt {2}$.

**Fig. 3.**
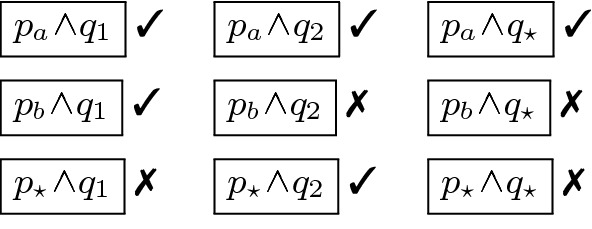
Cubes generated by the predicates $p_a, p_b, p_\star , q_1, q_2, q_\star$ generated from the policy in Fig. 1 and the result of querying Zelkova to check if the requests corresponding to each cube are granted access by the policy.

We can compute findings by enumerating all the *cubes* generated by the above predicates, and querying Zelkova to determine if the policy allows access to the requests described by the cube. For example, the above predicates would generate the cubes shown in Fig. 3. We omit trivially inconsistent cubes like $p_a \wedge p_b$ which correspond to the empty set of requests. Next to each cube, we show the result of querying Zelkova to determine whether the policy allows access to the requests described by the cube: ✓(resp. ✗) indicates requests are allowed (resp. denied).

Finally, we can translate each *allowed* cube into a finding, yielding five findings. While this set of findings is sound and precise, it suffers in two ways. First, real-world policies have many different fields, and hence, enumerating-and-querying each cube can be quite slow. Second, the result is not compact. The same information is more succinctly captured by the set of three findings in Sect. 2.1 which, for example, collapses the three findings in the top row to a single finding, “SrcVpc is vpc-a.”

***3: Stratified Abstraction.*** The chief difficulty with enumerating all the cubes *greedily* is that we end up eagerly *splitting-cases* on the values of fields when that may not be required. For example, in Fig. 3, we split cases on the possible value of OrgId even though it is irrelevant when SrcVpc is vpc-a. This observation points the way to a new algorithm where we *lazily* generate the cubes as follows. Our algorithm maintains a *worklist* of minimally refined cubes. At each step, we (1) ask Zelkova if the cube allows an access that is not covered by any of its refinements, (2) if so, we add it to the set of findings; and (3) if not, we refine the cube “point-wise” along the values of each field individually and add the results to the worklist. The above process is illustrated in Fig. 2.

- ***Level 1.*** The worklist is initialized with $\top \wedge \top$ which represents the cube where we *don’t care* about the value of either SrcVpc or OrgId, *i.e.* which represents *every* possible request. Zelkova determines that every access allowed by this cube and by the policy are covered by one of the refinements of this cube (the second level of the tree). Thus this $\top \wedge \top$ finding is not essential, and we can find more precise findings. We indicate this by the red shade and the ✗. Next, we *refine* the above cube point-wise, by considering the two sub-cubes $p_a \wedge \top$ and $p_b \wedge \top$ which respectively represent the requests where SrcVpc is either vpc-a or vpc-b (and OrgId could be any value), and, the two sub-cubes $\top \wedge q_1$ and $\top \wedge q_2$ which respectively represent the requests where OrgId is either o-1 or o-2 (and SrcVpc could be any value). These refined cubes are added to the worklist and considered in turn.
- ***Level 2.*** Zelkova determines that there are requests allowed by $p_a \wedge \top$ and $\top \wedge q_2$ which are not covered by any of their refinements, hence those are shaded green and have a ✓. However, Zelkova rejects $p_b \wedge \top$ and $\top \wedge q_1$ as anything allowed by them is allowed by one of their refinements. Now we further refine the rejected cubes, but can *omit* considering the cubes $p_a \wedge q_1$, $p_a \wedge q_2$ and $p_b \wedge q_2$ in the unshaded boxes, as each of those is covered or subsumed by one of the two accepted cubes.
- ***Level 3.*** Hence, we issue one last Zelkova query for $p_b \wedge q_1$ which indeed allows a request which is not covered by any of its refinements (as it has none). Finally, we gather the set of accepted cubes, *i.e.* those in the green shaded boxes, and translate those to the findings described in Sect. 2.1.

## Algorithm

Next, we formalize our algorithm for computing policy summaries and show how it yields findings that are sound and precise. In Sect. 4 we demonstrate how our algorithm yields compact results for real-world policies..

### Policies and Findings

***Requests.*** Let $K= \{k_1, \ldots , k_n\}$ be a set of *keys*. Let $V_k= \{v_1, \ldots \}$ be a (possibly infinite) set of *values* for the key $k$. A *request* $r$ a mapping from keys $k$ to values in $V_k$. For example, the request $r_1$ maps the keys ${\mathsf {Principal}}$, ${\mathsf {SrcIP}}$, and ${\mathsf {OrgID}}$ as:

$$\begin{aligned} r_1 = \{ {\mathsf {Principal}}\mapsto \mathtt{123:user/A},\ {\mathsf {SrcIP}}\mapsto \mathtt{192.0.2.3},\ {\mathsf {OrgID}}\mapsto \mathtt{o}\text {-}\mathtt{1} \} \end{aligned}$$

***Policies.*** A *policy* is a predicate on requests $p: r\rightarrow { Bool }$. The *denotation* of a policy $p$ is the set of requests it allows:

$$\gamma (p) \doteq \{ r\mid p(r) = True \}$$

***Predicates.*** A *predicate* is a map ${\phi }: V_k \rightarrow { Bool }$. The *denotation* of a predicate is the set of values that satisfy the predicate:

$$\gamma ({\phi }) \doteq \{ v\mid {\phi }(v) = True \}$$

We define a partial order on predicates, ${\phi }_1 \preceq {\phi }_2$ iff $\gamma ({\phi }_1) \subseteq \gamma ({\phi }_2)$. For example:

$$\begin{aligned}{{\phi }_{123}}(v)&\ \doteq \ ``v\text { is a principal in account 123''} \\ {{\phi }_{ua}}(v)&\ \doteq \ ``v\text { is } \texttt {user}\hbox {-}{} \texttt {a} \text { in account 123''} \\ {{\phi }_{ub}}(v)&\ \doteq \ ``v\text { is } \texttt {user}\hbox {-}{} \texttt {b} \text { in account 123''} \end{aligned}$$

Here we have ${{\phi }_{ua}}\preceq {{\phi }_{123}}$ and ${{\phi }_{ub}}\preceq {{\phi }_{123}}$ because users are a type of principal. The set of predicates must always contain $\top$ and must have the following property: for all ${\phi }_1$, ${\phi }_2$ either ${\phi }_1 \preceq {\phi }_2$, ${\phi }_2 \preceq {\phi }_1$, or $\gamma ({\phi }_1) \cap \gamma ({\phi }_2) = \emptyset$. This ensures the set of predicates for a given key can be tree-ordered.

***Findings.*** A *finding* $\sigma$ is a map from keys $K$ to predicates ${\varPhi }$. The *denotation* of a finding $\sigma$ is the set of requests where each key $k$ is mapped to a value $v$ in the denotation of $\sigma (k)$:

$$\gamma (\sigma ) \doteq \{ r\mid \forall k. r(k) \in \gamma (\sigma (k)) \}$$

We represent a finite set of findings as $\varSigma = \{\sigma _1, \ldots , \sigma _n\}$. The *denotation* of a set of findings is the union of the denotations the findings:

$$\gamma (\{\sigma _1,\ldots ,\sigma _n\}) \doteq \gamma (\sigma _1)\cup \cdots \cup \gamma (\sigma _n)$$

### Properties

Next, we formalize the key desirable properties of findings, *i.e.* that they be sound, precise, and compact, as *coverage*, *irreducibility*, and *minimality* respectively.

***Coverage.*** A set of findings $\varSigma$ *covers* a policy $p$ if $\gamma (p) \subseteq \gamma (\varSigma )$. For example, the set $\varSigma _1$ containing the two findings

$$\begin{aligned}\varSigma _1&\ \doteq \ \{ [ {\mathsf {SrcVpc}}\mapsto p_a, {\mathsf {OrgID}}\mapsto \top ], [ {\mathsf {SrcVpc}}\mapsto \top , {\mathsf {OrgID}}\mapsto q_2 ] \} \end{aligned}$$

corresponding to the green boxes on level 2 of Fig. 2, *does not* cover the policy from Fig. 1, as it excludes the request whose ${\mathsf {SrcVpc}}$ is $\mathtt {vpc}\text{- }\mathtt {b}$ and ${\mathsf {OrgID}}$ is $\mathtt {o}\text{- }\mathtt {1}$. However, $\varSigma _2$ below *does* cover the policy as it includes all requests that are granted access.

$$\begin{aligned} \varSigma _2&\ \doteq \ \varSigma _1 \cup \{ [ {\mathsf {SrcVpc}}\mapsto p_b, {\mathsf {OrgID}}\mapsto q_1 ] \} \end{aligned}$$

***Reducibility.*** A finding $\sigma$ refines another finding $\sigma '$, written $\sigma \sqsubseteq \sigma '$ if *for each* key $k$ we have $\sigma (k) \preceq \sigma '(k)$. A finding $\sigma$ refines a set of findings $\varSigma$, written $\sigma \sqsubseteq \varSigma$ if $\sigma$ refines *some* $\sigma ' \in \varSigma$. Note that $\sigma \sqsubseteq \sigma '$ implies $\gamma (\sigma ) \subseteq \gamma (\sigma ')$. We say that a finding $\sigma$ is *irreducible* for a policy $p$ if

$$\exists r\in \gamma (p) \cap \gamma (\sigma ).\ \forall \sigma ' \sqsubset \sigma .\ r\not \in \gamma (\sigma ').$$

That is, $\sigma$ is irreducible if it contains some request that is excluded by all its proper refinements. For example, the finding $[{\mathsf {SrcVpc}}\mapsto p_a, {\mathsf {OrgID}}\mapsto \top ]$ is irreducible as it contains a request $[{\mathsf {SrcVpc}}\mapsto \mathtt {vpc}\text{- }\mathtt {a}, {\mathsf {OrgID}}\mapsto \mathtt {o}\text{- }\mathtt {3}]$ that is excluded by its refinements $[{\mathsf {SrcVpc}}\mapsto p_a, {\mathsf {OrgID}}\mapsto q_1]$ and $[{\mathsf {SrcVpc}}\mapsto p_a, {\mathsf {OrgID}}\mapsto q_2]$. Note that irreducibility is inherently tied to the available predicates, ${\varPhi }$.

***Minimality.*** A set of findings $\varSigma$ is *minimal* if the denotation of each $\varSigma ' \subset \varSigma$ is strictly contained in the denotation of $\varSigma$. For example, the set

$$\{ [ {\mathsf {SrcVpc}}\mapsto p_a, {\mathsf {OrgID}}\mapsto \top ], [ {\mathsf {SrcVpc}}\mapsto p_a, {\mathsf {OrgID}}\mapsto q_1 ] \}$$

is *not* minimal as the subset containing just the first finding denotes the same set of requests, but, the set containing either finding individually *is* minimal.

### Algorithm

Given a policy $p$ and a finite set of partially ordered predicates ${\varPhi }$, our goal is to produce a minimal covering of $p$ comprising only irreducible findings.

***Access Oracle.*** Our algorithm is built using an *access oracle* that takes as input a policy $p$ and a finding $\sigma$ and returns $\mathsf {Some}$ iff some request described by $\sigma$ is allowed by $p$, and $\mathsf {None}$ otherwise.

$$\mathsf {CanAccess}(p, \sigma ) = \left. {\left\{ \begin{array}{ll} \mathsf {Some} &{} \text{ if }\ \gamma (\sigma ) \cap \gamma (p) \ne \emptyset \\ \mathsf {None} &{} \text{ if }\ \gamma (\sigma ) \cap \gamma (p) = \emptyset \end{array}\right. } \right. $$

**Fig. 4.**
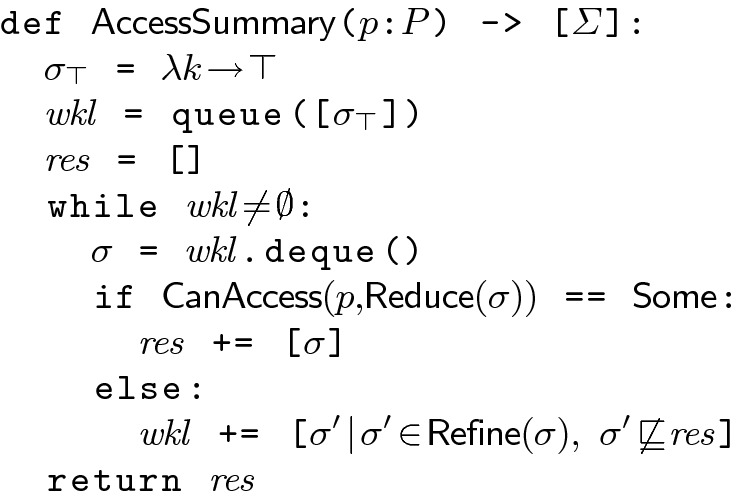
Algorithm to compute a minimal set of irreducible findings that cover policy $p$.

***Dominators.*** We define the *immediately dominates* set of ${\phi }\in {\varPhi }$ as the set of elements strictly smaller than ${\phi }$ but unrelated to each other:

$$\begin{aligned} \mathsf {idom}({\phi }) \doteq \{ {\phi }' \mid {\phi }' \prec {\phi }\ \text{ and }\ \forall {\phi }''. \lnot ({\phi }' \prec {\phi }'' \prec {\phi }) \} \end{aligned}$$

***Reducing a Finding.*** The procedure $\mathsf {ReducePred}$ (resp. $\mathsf {Reduce}$) takes as input a predicate ${\phi }$ (resp. finding $\sigma$) and strengthens it to *exclude* all the requests that are covered by the refinements of ${\phi }$ (resp. $\sigma$):
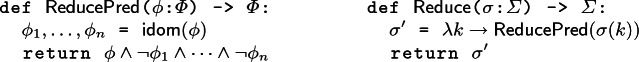


Intuitively, $\mathsf {Reduce}$ allows us to determine if a finding is irreducible.

#### Lemma 1

$\sigma$ is irreducible iff $\gamma (\mathsf {Reduce}(\sigma )) \cap \gamma (p) \ne \emptyset$.

***Refining a Finding.*** The procedure $\mathsf {Refine}$ takes as input a finding $\sigma$ and returns the set of findings obtainable by *individually* refining one value of $\sigma$.
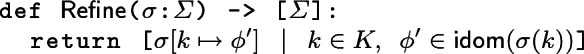


If a finding $\sigma$ is reducible, we will use $\mathsf {Refine}$ to *split* it into more precise findings.

#### Lemma 2

Let $\sigma$ be reducible for *p*. Then $\gamma (\sigma ) \cap \gamma (p) = \gamma (\mathsf {Refine}(\sigma )) \cap \gamma (p)$.

***Summarizing Access.*** The procedure $\mathsf {AccessSummary}$ (Fig. 4) takes as input a policy $p$ and returns a minimal set of irreducible findings $res$ that covers $p$. The procedure maintains a queue $wkl$ comprising a *frontier* of findings that are to be explored. The queue is initialized with the trivial finding $\sigma _\top$ that maps each key to $\top$. It then iteratively picks an element from the queue, checks if it is an irreducible finding, and if so, adds it to the result set $res$. If not, it computes the finding’s refinements and adds those to $wkl$. The process repeats till the queue is empty. The algorithm maintains three loop invariants: (1) $wkl \cup res$ covers $p$; (2) Each finding in $res$ is irreducible; (3) $res$ is minimal. Consequently, the algorithm terminates with a minimal set of irreducible findings that covers $p$. Note, the worklist is a queue so that if $\sigma _1 \sqsubset \sigma _2$ the algorithm will consider $\sigma _2$ before $\sigma _1$.

#### Theorem 1

Let $\varSigma = \mathsf {AccessSummary}(p)$. Then (1) $\varSigma$ covers $p$, (2) each $\sigma \in \varSigma$ is irreducible, and (3) $\varSigma$ is minimal.

## Implementation and Evaluation

The algorithm $\mathsf {AccessSummary}$ is implemented in the IAM Access Analyzer feature launched on Dec 2, 2019 [10]. The Zelkova tool [2] is used as the access oracle for the algorithm. Access Analyzer monitors the relevant resource policies in an account and re-runs the algorithm on any changes. Findings are presented to the user through a web console and through APIs. Users can *archive* findings that represent intended access to the resource. For unintended findings, Access Analyzer links to the relevant policy that users can edit to remove that access. Access Analyzer will automatically run on the changed policy and any findings that are no longer relevant will be set to a *resolved* state. By monitoring any existing or new *active* findings, users can ensure their polices grant only the intended access.

***Evaluation Metrics.*** We evaluate our algorithm along two dimensions: (1) “how efficient is the algorithm at generating findings?” and (2) “how effective are the generated findings at simplifying the complexity of a policy?”. As our algorithm solves a new problem, we do not have an external basis for comparison. Instead, we compare the algorithm against the state space it operates over. To this end, for each policy, we define the following measures:

- **size** is the size of the set of all possible findings for the policy.
- **findings** is the number of findings produced by the algorithm.
- **queries** is the number of SMT queries made by the algorithm.
- **runtime** is the total runtime of the algorithm.

Note that $\mathbf{findings} \le \mathbf{queries} \le \mathbf{size}$, as each query generates at most one finding and we query each possible finding at most once.

***Benchmarks.*** We randomly selected 1,387 policies from a corpus of in-use policies. As we are interested primarily in difficult policies, we filtered out all policies that had **size** less than 10. That left 165 policies. Each policy was evaluated on a 2.5 GHz Intel Core i7 with 16 GB of RAM. The runtime per finding ($\mathbf{runtime} / \mathbf{findings}$) was less than 430ms for all policies except one outlier at 2,267 ms. The 165 policies ranged in size from 56 to 810 lines of pretty-printed JSON with a median size of 91 lines.

**Fig. 5.**
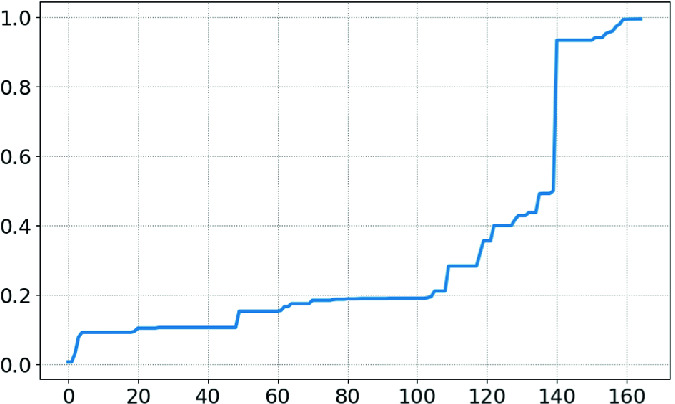
Actual findings vs. search space

**Fig. 6.**
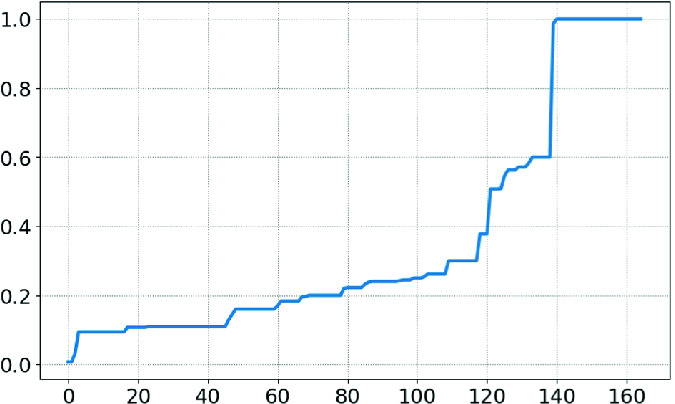
Actual queries vs. search space

***Results.*** Figures 5 and 6 show the number of findings and queries, respectively, compared to the overall search space. Both graphs are sorted to be monotonic, *i.e.* the *x*-axes are different. Figure 5 shows to what degree the findings simplify the policy, with smaller numbers being better. This measure will always be between 0 and 1 since $0 \le \mathbf{findings} \le \mathbf{size}$. We see that 85% of policies achieve a ratio of 0.5 or better, and 64% achieve a ratio of 0.2 or better. Figure 6 shows how efficient the algorithm is in exploring its state space, with smaller numbers being better. This measure is between 0 and 1 as $0 \le \mathbf{queries} \le \mathbf{size}$. The algorithm explores the entire search space for only 15% of the policies, with a median ratio of 0.22.

## Related Work

The majority of tools available for access policy analysis are based on log analysis or syntactic pattern matching, which are both imprecise (*i.e.* fail to account for the complex logic in AWS policies) and unsound (*i.e.* fail to check for all requests) and hence, can take months to discover that resources are susceptible to potentially unintended access. Most formal methods based work has focused on securing individual pieces of cloud infrastructure via low-level proofs of software correctness *e.g.* Ironclad [6]. Cloud Contracts [4] are requirements over network access control lists and routing tables. Cloud Contracts are verified using the SecGuru tool [7] that compares network connectivity policies using the SMT theory of bit vectors. In contrast, our work answers a larger question about the entire enterprise-level security posture using a series of Zelkova queries [2]. The Fireman system [11] shows how to use Binary Decision Diagrams to analyze access control lists (ACL) in firewall configurations. The ACL configuration language is more restricted than IAM’s and the tool is limited to a fixed set of queries about which accesses (packets) are allowed. Most closely related to our work is the Margrave system [9] which encodes firewall policies as propositional logic formulas, and then use SAT solvers to answer queries about the policies. Margrave introduces the notion of *scenario finding*, and shows how to produce an exhaustive set of scenarios that *witness* the queried behavior. The IAM policy language is significantly richer, and hence, enumerating scenarios is computationally intractable, which led us to the develop stratified abstraction as a means of summarizing policy semantics, thereby providing analysts comprehensive visibility into the accessibility of resources, helping detect misconfigurations, and ensuring that updates indeed fix the potential for unintended accesses.

## Footnotes

1 The finding “OrgId is o-2” also includes some requests that are not allowed, *e.g.* when SrcVpc is vpc-b.
